# Inhibitory effects of serum from sepsis patients on epithelial cell migration in vitro: a case control study

**DOI:** 10.1186/s12967-016-1110-7

**Published:** 2017-01-13

**Authors:** Henna Jaurila, Vesa Koivukangas, Marjo Koskela, Fiia Gäddnäs, Sirpa Salo, Johanna Korvala, Maija Risteli, Toni Karhu, Karl-Heinz Herzig, Tuula Salo, Tero I. Ala-Kokko

**Affiliations:** 1 Research Group of Surgery, Anesthesia and Intensive Care, Oulu University Hospital, P. O. Box 21, 90029 Oulu, Finland; 2 Cancer and Translational Medicine Research Unit, Faculty of Medicine, Medical Research Center Oulu, University of Oulu, P.O. Box 5281, 90014 Oulu, Finland; 3 Research Group of Biomedicine, Faculty of Biochemistry and Molecular Medicine, University of Oulu, P. O. Box 5000, Oulu, 90014 Finland; 4 Research Unit of Biomedicine, Faculty of Medicine and Biocenter of Oulu, University of Oulu, P.O. Box 5000, Oulu, 90014 Finland; 5 Department of Gastroenterology and Metabolism, Poznan University of Medical Sciences, Collegium Maius, Fredry 10, 61-701 Poznan, Poland; 6 Research Group of Oral Health Sciences, Oulu University Hospital, Medical Research Center Oulu, University of Oulu, P. O. Box 5000, Oulu, 90014 Finland

**Keywords:** Sepsis, Human serum, Migration, EGF, TNF-α, Exosome, HaCaT, In vitro

## Abstract

**Background:**

Sepsis delays wound re-epithelialization. In this study we explored the effect of human sepsis sera as well as the effects of cytokines, growth factors and exosomes of sepsis sera treated normal fibroblasts (NF) on keratinocyte migration and proliferation in vitro.

**Methods:**

Serum samples were taken on days 1, 4, and 9 from 44 patients diagnosed with severe sepsis, and from 14 matching healthy controls. We evaluated the effects of sepsis serum with or without TNF-α, EGF, EGF receptor inhibitor or exosomes of sepsis sera treated NF on human keratinocyte (HaCaT) proliferation (BrdU assay), viability (MTT assay), and migration (horizontal wound healing model). Cytokine levels of sepsis and healthy sera were measured by multiplex assay. Comparisons between groups were carried out using SPSS statistics and P < 0.05 was considered significant.

**Results:**

Severe-sepsis sera collected on days 1, 4, and 9 reduced keratinocyte proliferation by 6% (P = 0.005), 20% (P = 0.001), and 18% (P = 0.002), respectively, compared to control sera. Cell viability in cultures exposed to sepsis sera from days 4 and 9 was reduced by 38% (P = 0.01) and 58% (P < 0.001), respectively. Open-surface wounds exposed to sepsis sera from days 1 and 4 were larger than those exposed to sera from healthy controls (60 vs. 31%, P = 0.034 and 66 vs. 31%, P = 0.023, respectively). Exosomes of sepsis or healthy sera treated NF inhibited keratinocyte migration. We detected higher serum levels of cytokines TNF-α (5.7 vs. 0.7 pg/ml, P < 0.001), IL-6 (24.8 vs. 3.8 pg/ml, P < 0.001), IL-10 (30.0 vs. 11.9 pg/ml, P = 0.040), and VEGF (177.9 vs. 48.1 pg/ml, P = 0.018) in sepsis sera. Levels of EGF were significantly lower in sepsis sera than in that of healthy controls (6.5 vs. 115.6 pg/ml, P < 0.001). Sepsis serum supplemented with EGF 5 ng/ml and TNF-α in all concentrations improved keratinocyte migration.

**Conclusions:**

Keratinocyte viability, proliferation and migration were reduced in severe sepsis in vitro. Exosomes from NF added in healthy or sepsis serum media inhibited keratinocyte migration. Decreased levels of EGF in sepsis sera may partially explain the delay of wound healing with severe-sepsis patients. Increased levels of TNF-α in sepsis sera do not explain diminished keratinocyte migration.

## Background

Sepsis is defined as a dysregulated host response to outer pathogens leading to acute organ dysfunction [1, 2]. Skin is the main defensive barrier against outer pathogens. Disorders in skin function and wound healing during sepsis may lead to blistering and pressure ulcers that, in addition to surgery and invasive cannulations, compromise this defensive barrier [3]. Septic patients are prone to wound healing complications such as infections, delayed wound healing, fascial dehiscence and anastomotic leaks [4–9]. Septic patients’ predisposition to get spontaneous and iatrogenic wounds combined with impaired wound healing can lead to substantial functional and aesthetic, even life threatening problems. In our previous studies, skin collagen synthesis is proven to be diminished [10], the restoration of the epidermal barrier function to be delayed and wound blood flow increased in severe sepsis [8]. Wound re-epithelialization is delayed during sepsis, as demonstrated in rodent models [5, 7, 9] and in a human blister wound model [8]. Re-epithelialization is achieved via the migration and proliferation of keratinocytes from the edges of the wound. Cell migration is the rate-limiting event in the healing of skin wounds [11–13]. After injury the wound repair process is initiated immediately by the release of growth factors and cytokines from the serum, which bind to receptors expressed by their target cells, and co-ordinate the re-epithelialization [3, 11, 13–16]. However, knowledge about cell migration and proliferation in human systemic sepsis is currently limited; sepsis wound-healing studies have been conducted only in animals [5, 7, 9]. The role of cytokines involved in wound healing in sepsis is somewhat unclear. Exosomes are secreted membrane enclosed vesicles containing proteins like Epithelial Growth Factor Receptor (EGFR) and nucleic acids [17–20]. Previously, exosomes were thought to only eliminate waste proteins from the cell, but now they are known to participate in intercellular communication and the transfer of functional genetic information thereby influencing the immune system [21, 22]. The role of exosomes in disease pathogenesis is under investigation. There have been some studies about exosomes in sepsis or in wound healing [23–29]. However, the effect of exosomes in migrating septic wounds is unknown.

Here we hypothesized that sera from septic patients could reduce the viability, proliferation, and in vitro wound healing (horizontal migration) of human skin keratinocytes. In order to elucidate a possible mechanism related to delayed wound healing, we measured cytokine levels and analyzed the distinctions between sera collected from septic patients and from healthy controls. In addition, different concentrations of selected cytokines and exosomes of sepsis and healthy sera treated gingival fibroblasts were added to in vitro keratinocyte wounds in order to explore their influence on cell migration. We expected to see some differences between exosomes from sepsis and healthy sera treated fibroblasts in keratinocyte migration.

## Methods

### Patients

This prospective observational case–control study of wound healing in severe sepsis was conducted in a 12-bed mixed-type intensive care unit (ICU) in Oulu University Hospital, Finland. This investigation is a substudy of an earlier work that examined serum markers of collagen synthesis and degradation in severe sepsis [30]. The inclusion criterion was diagnosis of severe sepsis according to the American College of Chest Physicians/Society of Critical Care Medicine [1]. The exclusion criteria were: age under 18 years, malignancy, surgery not related to sepsis, surgery during the preceding 6 months, bleeding disorder, chronic hepatic or renal failure, and immunosuppressive or cortisone treatment not related to sepsis. Patients entered the study when the diagnosis of severe sepsis was met and within 48 h of the first identification of organ dysfunction. Patients were treated according to normal ICU protocol and current severe sepsis guidelines [31]. The study protocol was approved by The Regional Ethics Committee of the Northern Ostrobothnia Hospital District and written informed consent was obtained from each patient or their next of kin. The following information was collected from all patients: age, gender, type of ICU admission (medical or surgical), prevalence of septic shock, severity of underlying diseases on admission as assessed with the Acute Physiology and Chronic Health Evaluation II (APACHE II), and development of daily organ dysfunctions assessed with the daily Sequential Organ Failure Assessment (SOFA). Length of stay in the ICU and 30-day mortalities were recorded. Fourteen healthy sex- and age-matched volunteers served as controls.

### Blood samples

Serum samples were collected after the first identification of sepsis-induced organ dysfunction. Samples were taken on days 1, 4, and 9 or until the patient was transferred to another unit/hospital or died. Serum samples from healthy controls were obtained once. The serum samples were immediately frozen and stored at −70 °C. In the cell-migration and proliferation experiments, individual serum samples were filtered and pooled using serum from each patient for final serum concentrations of 1% (in migration tests) or 10% (in proliferation tests) in serum free cell-culture medium. This strategy resulted in serum pools of 44 sepsis-serum samples on study day 1, 36 samples on day 4, 22 samples on day 9, and a single pool of 14 healthy control serum samples. As an experimental control, we used 1 or 10% fetal bovine serum (FBS; Invitrogen, Carlsbad, CA, USA). Cytokine analysis employed undiluted, individual serum samples.

### Cell lines

Human adult low-calcium high-temperature (HaCaT) cells are a spontaneously transformed human epithelial cell line from adult skin and maintain full epidermal differentiation capacity. These keratinocytes are immortalized and have unlimited growth potential, but still they are non-tumorigenic [32]. In our study, HaCaT cells were maintained in Dulbecco’s modified Eagle’s medium (DMEM) (Sigma-Aldrich, St. Louis, MO, USA) supplemented with 10% heat-inactivated FBS, 100 U/ml penicillin, 100 μg/ml streptomycin, 50 μg/ml ascorbic acid, 250 ng/ml fungizone, and 1 mM sodium pyruvate (all from Sigma-Aldrich). Cells were derived from the freeze-down batch, which was thawed and grown to confluence in a 175-cm^2^ flask. The cells were incubated at 37 °C, in 5% CO_2_, and 95% humidity. The number of passages in all cell lines was less than 23.

### Assay of keratinocyte (HaCaT) proliferation

To evaluate cell proliferation, HaCaT cells (10^5^ cells per well) were seeded, and cultured 24 h on 96-well plates. In each well, 100 µL of 10% test serum cocktail were pipetted onto HaCaT cells. After 24 h of incubation, cell proliferation was quantified via a colorimetric immunoassay of the incorporation of the thymidine analog 5-bromo-2′-deoxyuridine (BrdU) during DNA synthesis according to the manufacturer’s instructions (Roche Diagnostics, Basel, Switzerland). Absorbance values were measured with a Victor3 V 1420 Multilabel Plate Counter (PerkinElmer, Waltham, MA, USA) at a wavelength of 355 nm. Assays were performed in triplicate and mean values were recorded.

### Assay of keratinocyte (HaCaT) viability

HaCaT cells (10^5^ cells per well) were seeded and cultured 24 h on 96-well plates. In each well, 100 µL of 10% test serum cocktail were pipetted onto the cells, which were then incubated for 48 h. Cell viability was assayed with 3-[4,5-dimethylthiazol-2-yl]-2-5-diphenyl tetrazolium bromide (MTT) according to the manufacturer’s instructions (Sigma-Aldrich). The number of living cells, evaluated via mitochondrial dehydrogenase activity, was measured with a Victor3 V 1420 Multilabel Plate Counter (PerkinElmer) at a wavelength of 544 nm. Assays were performed in triplicate and mean values were recorded.

### Horizontal wound healing assay

Epithelial wound healing was investigated in vitro using an assay in which the wounds were made by plating the counted cells into commercial inserts (Ibidi GmbH, Munich, Germany) instead of scratching. In the migration tests, 1 × 10^5^ or 2 × 10^5^ cells per well were seeded and cultured on a 24-well plate in culture inserts. The silicone insert was removed after 24 h of incubation so that the resulting cell patch was split into two parts separated by a 500 µm cell-free zone. Cell-culture medium was replaced with serum free medium supplemented with 1% test serum samples. After 0, 12, 24, 36, and 48 h of incubation, cell migration (reduction in wound surface area) was recorded with a digital inverted microscope (Evos fl AMF-4302, AMG Life Technologies, Carlsbad, CA, USA) and an in vivo microscope camera (ICX285AL monochrome CCD, Sony, Tokyo, Japan). The plates were incubated at 37 °C between measurements. Open-wound areas on the digital images were measured using ImageJ [33]. There were 4–8 wounds in each group. We calculated the mean value of the remaining cell-free area at each time point in every group as well as the percentage by which the initial gap width decreased at each time point.

### Exosome isolation and horizontal wound healing assay with exosomes

Human normal gingival fibroblasts (NF) [34] were used to isolate exosomes. Cells were maintained in DMEM supplemented with 10% heat-inactivated FBS, 1 mM sodium-pyruvate, 100 U/ml penicillin, 100 µg/ml streptomycin, 50 µg/ml ascorbic acid and 250 ng/ml fungizone (all from Sigma-Aldrich) and incubated at 37 °C in 5% CO_2_. For exosome isolation 500,000 cells were seeded in 175 cm^2^ flasks and cultured 24 h in normal culture medium. The cells were washed once with phosphate buffered saline. Serum free medium supplemented with a 1% test serum pool of healthy or day one sepsis sera was added. Media was collected after 48 h, and centrifuged at 300×*g* for 2 min to remove dead cells. The supernatant was collected and stored at −70 °C until exosome isolation. Conditioned medium was thawed and ultracentrifuged at 10,000×*g* for 90 min at +4 °C in a swinging bucket TH-641 rotor (Thermo Fisher Scientific Inc., Waltham, MA, USA). The supernatant was removed to a fresh tube leaving 500 µl in the bottom of the previous tube. The supernatant was ultracentrifuged again at 100,000×*g* for 90 min to pellet exosomes. The supernatant was removed except for 200 µl and the pellet was re-suspended into this remaining supernatant. The protein concentration was measured with a DC Protein assay (Bio-Rad). In the horizontal wound-healing assay 20 and 50 µg/ml of exosomes in serum free media were used both in healthy and sepsis groups. As controls we used 1% healthy and day one sepsis sera in serum free media and the horizontal wound healing assays were performed as described above. There were seven to eight wounds in each group.

### Assays of cytokines and growth factors

The levels of serum cytokines and growth factors were measured by multiplex assay [35, 36] with a Milliplex Human Cytokine/Chemokine Magnetic Bead Panel (Millipore Corporation, Billerica, MA, USA) and a Bio-Plex 200 System (Bio-Rad Laboratories Pty Ltd, Hercules, CA, USA). Assays were performed according to the manufacturer’s instructions, as described previously [37]. Assay conditions were pre-optimized, standardized, and controlled to ensure optimal reproducibility. Results were calculated with BioPlex Manager Software 6.0 (Bio-Rad Laboratories). Serum levels of interleukin (IL)-4, IL-6, IL-10, tumor necrosis factor α (TNF-α), basic fibroblast growth factor (bFGF), vascular endothelial growth factor (VEGF), and epithelial growth factor (EGF) were compared between samples from sepsis patients and healthy controls. The levels of cytokines and growth factors on day four were evaluated; at day four, the difference in keratinocyte migration and proliferation was most striking between model wounds exposed to sepsis sera or to healthy sera.

### Horizontal wound healing assay with EGF, TNF-α and EGFR inhibitor

To explore keratinocyte migration, the horizontal wound healing assay was used in which 1% healthy or sepsis serum pools were supplemented with 5, 10 or 50 ng/ml of EGF or TNF-α (both from ProSpec, East Brunswick, NJ, USA) as well as 1, 10 or 50 µg/ml of EGFR inhibitor (Erbitux (cetuximab) 5 mg/ml, Merck, Germany). In this experiment, 1% healthy and sepsis serum samples without supplements served as controls. The migration test was performed three times and the number of wounds was between 4 and 8 in each group.

### Statistical analysis

Data were entered into an SPSS database for analysis (SPSS version 21, IBM SPSS Statistics, Chicago, IL, USA). Summary measurements for variables were expressed as the median with 25th–75th percentiles or as the mean with standard deviation (SD). Comparisons between groups were performed using the independent-samples *t* test and the Mann–Whitney U test. Two-tailed P values were reported when possible. Differences were considered significant at P < 0.05.

## Results

### Patients

Between May 2005 and December 2006, 1361 patients were admitted to the ICU at Oulu University Hospital. Of these patients, 238 had severe sepsis and 66 met the inclusion criteria for this study. Written informed consent was obtained from 44 patients or their next of kin. Patient demographics have been presented previously [30]. Most of the 44 patients were male (66%) and there were 33 survivors (75%) on day 30 (Table 1). The control group consisted of 14 healthy age- and sex-matched volunteers, eight of them men (57%). The median age of the control group was 61 years (25th–75th percentile, 56–69 years).

**Table 1:** Summary demographics of the 44 study patients with severe sepsis

	Severe sepsis (n = 44)
Male sex, n (%)	29 (66%)
Age, years	63 (56–71)
Surgical admission, n (%)	25 (57%)
Septic shock, n (%)	40 (91%)
APACHE II score on admission, points	26 (22–31)
SOFA score on admission, points	8 (6–12)
Length of stay in the ICU, days	7 (4–12)
30-day mortality, n (%)	11 (25%)

Variables are presented as frequencies with percentages or as medians with 25th to 75th percentiles

*Apache II* acute physiology and chronic health evaluation II score

*Sofa* sequential organ failure assessment

### Keratinocyte proliferation and viability is diminished with sepsis sera

**Fig. 1:**
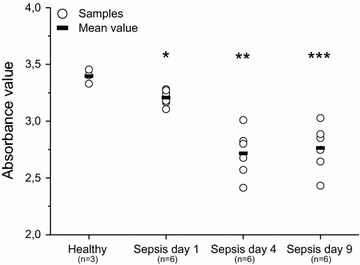
Keratinocyte (HaCaT) proliferation after exposure to healthy and sepsis sera (BrdU assay). Statistically significant differences in mean absorbance values between cells treated with sepsis and healthy sera are marked with* asterisks* (*P = 0.005, **P = 0.001, ***P = 0.002)

**Fig. 2:**
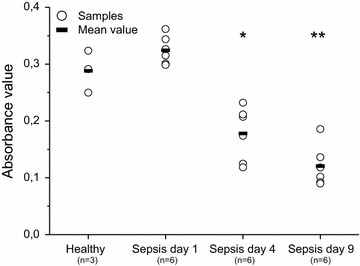
Keratinocyte viability after exposure to healthy and sepsis sera (MTT assay). Statistically significant differences in mean absorbance values between cells treated with sepsis sera and cells treated with healthy sera are marked with* asterisks* (*P = 0.01, **P < 0.001)

In order to measure whether sepsis serum contains substances that affect cell proliferation and viability, HaCaT cells were incubated in the presence of healthy and sepsis serum. In the BrdU proliferation assay, the proliferation of cells exposed to day one sepsis serum was 6% lower (P = 0.005) compared with cells exposed to healthy serum, 20% lower (P = 0.001) in day 4 serum, and 18% lower (P = 0.002) in day 9 serum (Fig. 1). The MTT cell viability assay indicated that cells incubated with days 4 and 9 sepsis serum were significantly less viable than cells treated with healthy serum (38%, P = 0.01 and 58%, P < 0.001, respectively) (Fig. 2). Day-one viability was slightly increased by 13%; but this difference was not statistically significant (P = 0.115) (Fig. 2).

### Sepsis delays keratinocyte migration in the early days of the disease

**Fig. 3:**
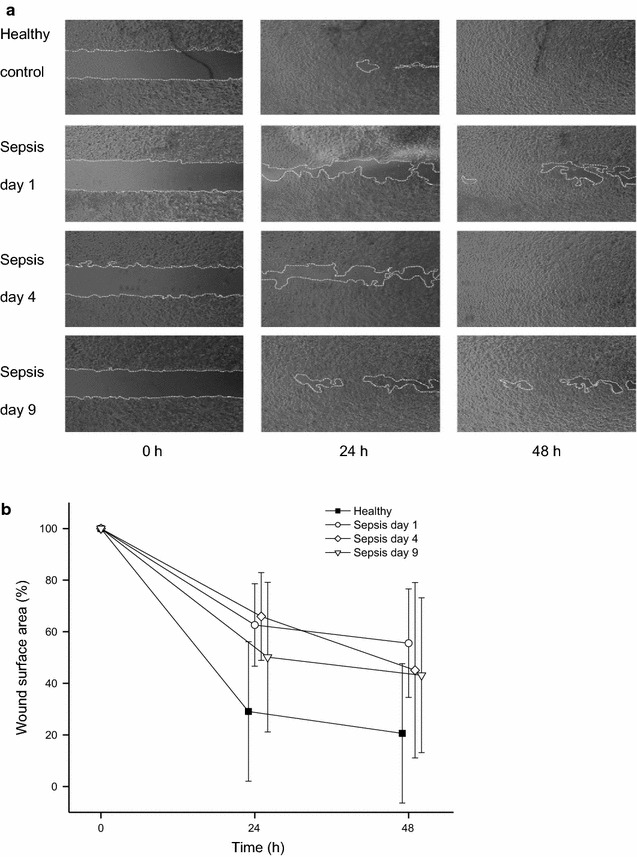
Keratinocyte migration after exposure to healthy and sepsis sera (horizontal wound-healing assay). **a** An example of the wound surface area calculation from the microscopy images of cell migration in wounds after 0, 24 and 48 h exposure to healthy or sepsis sera. *White dotted lines* mark the open wound area. The open surface area was measured using ImageJ. **b** Closure of open wound areas of keratinocytes at 0, 24 and 48 h (% of 0 h area). The data present means with standard deviations from eight scratch wounds incubated with day 1, 4, and 9 sepsis sera and means with standard deviations from four healthy sera control wounds

Effect of the sepsis serum on cell migration was tested using a wound-healing assay (Fig. 3a). The open wound surface area in models exposed to healthy sera was 31% of the original wound gap (SD 27%) at 24 h and 22% (SD 27%) at 48 h. Wounds cultured with sepsis sera from days 1 and 4 had significantly larger wound surface areas (60%, SD 16%, P = 0.034 and 66%, SD 17%, P = 0.023, respectively) at 24 h, than wounds cultured with healthy sera (Fig. 3b). Wounds treated with sepsis sera from day nine did not significantly differ from those treated with healthy sera at 24 h (50%, SD 29%, P = 0.297). However, because the culture media was not changed, the cells started to starve. So, most likely, although the tendency remained the same, no statistically significant differences between healthy and sepsis sera samples were seen after 48 h incubation (day 1: 53%, SD 21%, P = 0.053; day 4: 46%, SD 34%, P = 0.263; day 9: 43%, SD 30%, P = 0.273) (Fig. 3b).

### Exosomes derived from healthy or sepsis sera treated fibroblasts reduce keratinocyte migration

**Fig. 4:**
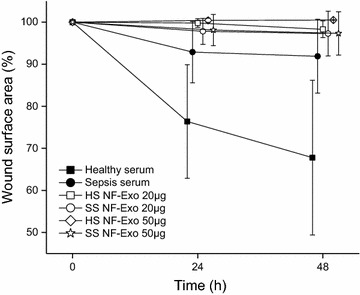
Keratinocyte migration in wounded monolayer after exposure to exosomes. Exosomes derived from healthy sera (HS NF-Exo) and sepsis sera (SS NF-Exo) treated normal fibroblasts were used in 20 or 50 µg/ml concentrations. Wound area (%) reduction was followed 48 h and data are presented as means with standard deviations from seven wounds per group

In the horizontal wound healing assay, we used 20 and 50 µg/ml of both healthy and sepsis exosomes in serum-free media. As controls, we used 1% healthy or sepsis sera. Keratinocytes in controls migrated as in previous experiments (Fig. 4). Migration in exosome treated wounds was significantly reduced compared to control wounds. Wounds with 20 or 50 µg/ml exosomes from healthy serum migrated significantly less than wounds with healthy control serum at 24 h (P = 0.018 and P = 0.015, respectively) and at 48 h (P = 0.025 and P = 0.021, respectively). Also wounds with 50 µg/ml exosomes from sepsis day 1 serum had a significant reduction in migration compared to sepsis control serum at 24 and 48 h (P = 0.027 and P = 0.037, respectively). Wounds with 20 µg/ml exosomes from sepsis day one serum migrated less compared to control serum either at 24 or 48 h, but the difference was not statistically significant (P = 0.083 and P = 0.132, respectively).

### Cytokine level differences between healthy and sepsis sera

At day 4 the difference in keratinocyte proliferation and migration was most notable between model wounds exposed to sepsis and healthy sera. To search for possible factors in sera that could explain this difference, we analyzed the levels of cytokines and growth factors in day four serum by multiplex assay. Sera collected on day four from patients with severe sepsis harbored significantly higher concentrations of TNF-α (5.7 vs. 0.7 pg/ml, P < 0.001), IL-6 (24.8 vs. 3.8 pg/ml, P < 0.001), and IL-10 (30.0 vs. 11.9 pg/ml, P = 0.040) than sera collected from healthy controls (Table 2). Of the growth factors, VEGF levels were higher (177.9 vs. 48.1 pg/ml, P = 0.018) and EGF levels were lower (6.5 vs. 115.6 pg/ml, P < 0.001) in severe sepsis serum than in healthy serum (Table 2). There were no significant differences in the levels of IL-4 (5.2 vs. 13.7 pg/ml) or basic fibroblast growth factor (32.2 vs. 21.9 pg/ml) between sepsis and control sera (Table 2).

**Table 2:** Cytokine levels (pg/ml) in sera from patients with severe sepsis (day four) and healthy sera

Cytokine	Sepsis sera	Healthy control sera	P value
EGF	6.5 (14.5)	115.6 (114.1)	<0.001
TNF-α	5.7 (4.9)	0.7 (0.2)	<0.001
IL-6	24.8 (20.6)	3.8 (5.4)	<0.001
VEGF	177.9 (185.7)	48.1 (32.2)	0.018
IL-10	30.0 (49.7)	11.9 (16.3)	0.040
IL-4	5.2 (9.7)	13.7 (24.9)	0.352
bFGF	32.2 (37.7)	21.9 (13.6)	0.810

Data are expressed as mean values and SD

### Sepsis serum supplemented with TNF-α and EGF enhance keratinocyte migration

**Fig. 5:**
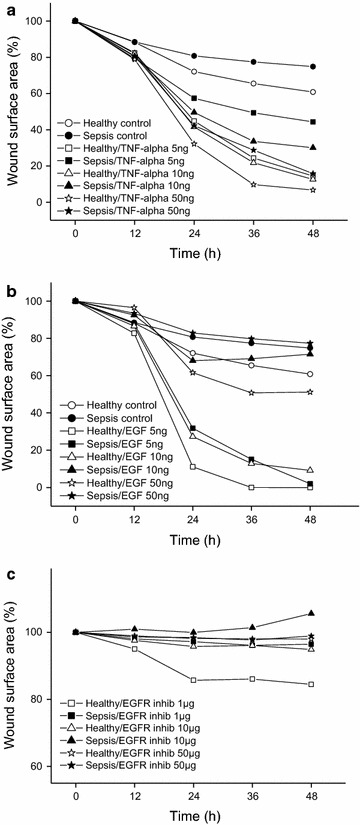
Keratinocyte migration after exposure to healthy and sepsis sera supplemented with cytokines and EGFR inhibitor. Migration was studied using a horizontal wound-healing assay and the open wound area (%) was measured by ImageJ every 12 h until 48 h. The data present means from four to eight wounds in every group.* Graphs* show results from control and test serums containing **a** 5–50 ng/ml TNF-α **b** 5–50 ng/ml EGF **c** 1–50 µg/ml EGFR inhibitor (cetuximab)

We conducted a wound-healing assay with healthy and sepsis sera containing different concentrations of TNF-α or EGF or EGF receptor inhibitor cetuximab. All concentrations of TNF-α improved cell migration in wounds, both those cultured in healthy and sepsis sera (P < 0.01 in all concentrations) (Fig. 5a). Furthermore, 5 or 10 ng/ml of EGF in healthy serum significantly enhanced keratinocyte migration (P = 0.001), but the addition of 50 ng/ml EGF did not (P = 0.768) have a similar effect (Fig. 5b). Sepsis serum with 5 ng/ml EGF significantly (P = 0.001) improved cell migration. Higher concentrations (10 and 50 ng/ml) of EGF in sepsis serum did not have a significant impact on migration. All concentrations of the EGFR inhibitor cetuximab both in healthy or sepsis sera, significantly impaired keratinocyte migration compared to control serum (Fig. 5c). P values are represented in Table 3.

**Table 3:** Serum supplemented with TNF-α/EGF/EGFR inhibitor, open wound surface area (%) of the original at 48 h

Serum	Cytokine	Amount of cytokine	Number of wounds	Mean % (SD)	P value
Healthy (control)	−	−	8	61 (15)	
Sepsis (control)	−	−	8	75 (20)	
Healthy	TNF-α	5 ng/ml	8	14 (15)	<*0.001*
Sepsis	TNF-α	5 ng/ml	7	44 (19)	*0.009*
Healthy	TNF-α	10 ng/ml	7	13 (15)	<*0.001*
Sepsis	TNF-α	10 ng/ml	5	30 (20)	*0.002*
Healthy	TNF-α	50 ng/ml	6	7 (16)	*0.002*
Sepsis	TNF-α	50 ng/ml	5	16 (18)	*0.005*
Healthy	EGF	5 ng/ml	6	0 (0)	*0.001*
Sepsis	EGF	5 ng/ml	8	2 (5)	*0.001*
Healthy	EGF	10 ng/ml	8	9 (17)	*0.001*
Sepsis	EGF	10 ng/ml	6	72 (57)	0.301
Healthy	EGF	50 ng/ml	4	51 (59)	0.768
Sepsis	EGF	50 ng/ml	6	77 (39)	0.887
Healthy	EGFr inhibitor	1 µg/ml	7	84 (24)	*0.037*
Sepsis	EGFr inhibitor	1 µg/ml	7	96 (7)	*0.008*
Healthy	EGFr inhibitor	10 µg/ml	5	95 (11)	*0.001*
Sepsis	EGFr inhibitor	10 µg/ml	6	106 (10)	*0.005*
Healthy	EGFr inhibitor	50 µg/ml	7	95 (4)	*0.001*
Sepsis	EGFr inhibitor	50 µg/ml	7	99 (2)	*0.001*

Healthy serum samples are compared to healthy control serum and sepsis serum samples to sepsis control serum. Significant P values are in italic

## Discussion

In this study, we explored the growth of human keratinocytes (HaCaT) in vitro using sera from severe sepsis patients and healthy controls. We observed that sepsis serum reduced keratinocyte viability, proliferation and migration. Additionally, exosomes excreted from normal fibroblasts treated with sepsis or healthy sera decreased keratinocyte migration. Furthermore, sepsis sera compared to healthy sera contained higher levels of TNF-α, IL-6, IL-10 and VEGF but a lower concentration of EGF. There were no significant differences in levels of bFGF or IL-4 between sepsis and control sera. All concentrations of TNF-α improved cell migration both in healthy sera and sepsis sera wounds. Low concentrations of EGF in healthy or sepsis sera significantly promoted keratinocyte migration, but migration was suppressed by blockade of the EGF receptor by cetuximab.

Considering the prevalence and economical effects of wound problems in septic patients, wound healing in sepsis is a poorly studied subject. To our knowledge, this is the first report presenting in vitro observations of the retarding effect of human sepsis serum on keratinocyte viability, migration, and proliferation. There are a few animal studies suggesting wound healing is impaired in sepsis [5, 7, 9]. This study is also a continuation to our previous research of human abdominal skin blister wounds, in which we showed that the restoration of epidermal barrier function was lower in patients with severe sepsis than in healthy controls [8].

The role of exosomes in intercellular signaling of skin tissue has recently been analyzed in animal models. Human fibroblast derived exosomes promoted keratinocyte proliferation, migration and wound closure in diabetic mice [38]. Similarly, rat burn wounds treated with mesenchymal stem cell (MSC) derived exosomes enhanced proliferation of skin cells and wound re-epithelialization [29]. We expected to see differences in keratinocyte migration between wounds incubated with exosomes from NF cultured in sepsis or healthy sera media. However, exosomes from NF cultured under both conditions clearly prevented the migration of keratinocytes. One explanation could be the resting state of these locally established NFs. Other studies have shown that with the help of exosomes, transplanted stem and progenitor cells use paracrine signaling to modify recipient cell protein production and gene expression in response to local environmental factors thus accelerating wound healing [20, 29, 39–43]. Thus exploring the contents of exosomes and using exosomes from more dynamic cells, such as MSCs, in both 2D and 3D cultures might give a broader perspective of their role in keratinocyte migration during the wound healing process.

Our results show that the lower amount of EGF is associated with the reduced cell proliferation, viability and migration of keratinocytes incubated with sepsis serum compared to healthy serum. Members of the EGF and FGF families as well as hepatocyte and insulin-like growth factors play a leading role in skin epithelialization during wound healing [13, 44]. Independent of the stimulant, keratinocyte migration seems to be universally conveyed through the EGF receptor; signaling through EGFR promotes keratinocyte migration in vitro [45]. Heparin-binding EGF-like growth factor (HB-EGF) accelerated keratinocyte migration, rather than proliferation in skin wound healing in a mouse model and seemed to be the predominant growth factor in epithelialization [46]. Similarly, the expression of HB-EGF in human keratinocytes triggered a migratory phenotype in partial-thickness wounding of human skin [47]. Application of EGF on wounds in vitro and in vivo had beneficial effects on skin wound healing [48, 49].

Sepsis sera contained higher levels of TNF-α, IL-6, IL-10 and VEGF compared to controls. TNF-α and IL-6 can indirectly induce keratinocyte migration via stimulating production of pro-mitogenic FGF-7, also known as keratinocyte growth factor, from fibroblasts [50]. However, according to our experiments the higher level of TNF-α in sepsis serum is not the reason for impaired keratinocyte migration and wound healing, in contrast to previous studies [51, 52]. Our research supports the suggestion of Sommer et al. [9] that normal TNF-α concentration locally enhances wound repair in sepsis. TNF-α is the primary inflammatory mediator in sepsis as it regulates other downstream cytokines such as IL-6 and IL-10 [53]. IL-6 participates only indirectly in keratinocyte migration: it mainly promotes collagen deposition and angiogenesis in cutaneous wound healing [54–57]. VEGF and its receptor primarily induce angiogenesis but have some influence on keratinocyte migration and proliferation as well [13, 16]. IL-10 has positive effects on wound closure, granulation tissue formation and neovascularization mainly because it improves VEGF expression [58]. Fibroblast growth factor levels are elevated in acute wound fluid and especially bFGF increases keratinocyte motility in re-epithelialization [16]. In our study we could not detect any significant difference in serum concentrations of bFGF between sepsis patients and controls, which suggests that bFGF has a minor role in keratinocyte migration in sepsis. IL-4 participates in normal wound healing by stimulating extracellular matrix synthesis [59] but does not seem to be involved in keratinocyte migration.

As stated earlier, epithelial wound healing in sepsis is affected by a complex mixture of various interacting signaling molecules. To better understand the mechanisms behind impaired wound healing in sepsis, further extensive studies are needed.

## Conclusions

In this study we show that human keratinocyte migration, proliferation, and viability were decreased in cultures treated with serum from patients with severe sepsis. Exosomes derived either from healthy or sepsis sera treated fibroblasts inhibited keratinocyte migration. Sepsis sera supplemented with EGF improved and EGF receptor inhibition significantly reduced keratinocyte migration both in healthy and sepsis wounds. Taken together, the net effect of serum on keratinocytes depends on the balance and interplay of various mediators.

## Abbreviations

APACHE II: acute physiology and chronic health evaluation II
bFGF: basic fibroblast growth factor
BrdU: 5-bromo-2’-deoxyuridine
DMEM: Dulbecco’s modified Eagle’s medium
EGF: epithelial growth factor
EGFR: epithelial growth factor receptor
FBS: fetal bovine serum
FGF: fibroblast growth factor
HaCaT: human adult low-calcium high-temperature keratinocytes
ICU: intensive care unit
IL: interleukin
MTT: 3-[4, 5-dimethylthiazol-2-yl]-2-5-diphenyl tetrazolium bromide
NF: normal fibroblast
SD: standard deviation
SOFA: sequential organ failure assessment
TNF-α: tumor necrosis factor α
VEGF: vascular endothelial growth factor
